# Fabrication of tissue‐engineered cell sheets by automated cell culture equipment

**DOI:** 10.1002/term.2968

**Published:** 2019-11-14

**Authors:** Ayako Nishimura, Ryota Nakajima, Ryo Takagi, Guangbin Zhou, Daisuke Suzuki, Masaharu Kiyama, Takayuki Nozaki, Toshiyuki Owaki, Tomomi Takahara, Shigeru Nagai, Taku Nakamura, Masakazu Sugaya, Koichi Terada, Yumiko Igarashi, Hiroko Hanzawa, Teruo Okano, Tatsuya Shimizu, Masayuki Yamato, Shizu Takeda

**Affiliations:** ^1^ Research & Development Group| Hitachi, Ltd. Hatoyama Japan; ^2^ Institute of Advanced Biomedical Engineering and Science Tokyo Women's Medical University Shinjuku‐ku Japan

**Keywords:** automated cell culture equipment, cell sheet, closed cell culture vessel and circuit module, oral mucosal epithelial cells, regenerative medicine, temperature‐responsive culture surface

## Abstract

Most cells for regenerative medicine are currently cultured manually. In order to promote the widespread use of regenerative medicine, it will be necessary to develop automated culture techniques so that cells can be produced in greater quantities at lower cost and with more stable quality. In the field of regenerative medicine technology, cell sheet therapy is an effective tissue engineering technique whereby cells can be grafted by attaching them to a target site. We have developed automated cell culture equipment to promote the use of this cell sheet regenerative treatment. This equipment features a fully closed culture vessel and circuit system that avoids contamination with bacteria and the like from the external environment, and it was designed to allow 10 cell sheets to be simultaneously cultured in parallel. We used this equipment to fabricate 50 sheets of human oral mucosal epithelial cells in five automated culture tests in this trial. By analyzing these sheets, we confirmed that 49 of the 50 sheets satisfied the quality standards of clinical research. To compare the characteristics of automatically fabricated cell sheets with those of manually fabricated cell sheets, we performed histological analyses using immunostaining and transmission electron microscopy. The results confirmed that cell sheets fabricated with the automated cell culture are differentiated in the same way as cultures fabricated manually.

## 1 INTRODUCTION

Regenerative medicine refers to an innovative medical treatment that uses cells to restore the function of living tissues and organs damaged by various causes. Unlike organ transplantation, it is able to overcome problems such as donor shortages. Cells for regenerative medicine are cultured manually by skilled technicians in cell‐culture clean rooms that comply with Good Manufacturing Practice (GMP) standards by maintaining a high level of cleanliness to avoid bacterial contamination. To make regenerative medicine more available, it is essential to facilitate mass production, reduce costs, and stabilize the quality of cell and tissue production. It is expected that automated cell culture techniques will be developed by combining advanced technologies. There are two types of automated cell culture system: one is a sealed‐chamber culture system using a robotic arm; the other is a sealed‐vessel culture system (Kino‐oka & Taya, 2009). The sealed‐chamber culture system requires sterilization of the entire interior of the device during batch changes. On the other hand, the sealed‐vessel culture system only has a small volume that requires sterilization. For this reason, we chose a sealed‐vessel culture system and developed closed‐system automated cell culture equipment that has excellent sterility qualities. The main advantage of this equipment is its closed culture vessel/circuit system that completely avoids contamination by bacteria and the like from the external environment (T. Kobayashi, Kan, Nishida, Yamato, & Okano, 2013; Matsumoto et al., 2019; Nakajima et al., 2015; Shu et al., 2016).

After cells have been expanded on a culture dish, they are usually treated with an enzyme such as trypsin or dispase so they can be detached from the culture dish. However, since enzymes can destroy adhesive proteins and important cell membrane proteins, this process could cause drastic changes to the structure and function of the cells. In contrast, a cell sheet cultured on a dish with a temperature‐responsive surface can be harvested from the culture dish without using any enzymes and without impairing its structure and function simply by reducing the temperature from 37°C to 20°C (Okano, Yamada, Sakai, & Sakurai, 1993; Yamada et al., 1990). Since this means the cell sheet can be harvested intact together with the intra‐cellular binding protein and the adhesive protein, it can be transplanted very efficiently, and has excellent therapeutic effects.

This technique has a very wide range of applications, and can be used to fabricate cell sheets from many types of cell, including corneal epithelial cells, oral mucosal epithelial cells, fibroblasts, and nasal mucosal cells (Hayashi et al., 2010; Kanzaki et al., 2008; Nishida et al., 2004; Yamamoto et al., 2017). Myoblast cell sheets have been put to practical use, and clinical trials have also been conducted on other cell sheets including chondrocytes (Sato, Yamato, Hamahashi, Okano, & Mochida, 2014; Sawa et al., 2015). Clinical studies in Japan and Sweden have confirmed that cell sheets of the oral mucosal epithelial cells targeted in this study are effective at preventing stenosis following the excision of early‐stage esophageal cancer; clinical trials are currently under way in Japan (S. Kobayashi et al., 2014; Ohki et al., 2012).

Using this automated cell culture equipment, we fabricated 50 sheets of oral mucosal epithelial cells in five automated culture tests in this study, and we evaluated the quality of these sheets according to the cell sheet quality standards for clinical research that apply to the use of cell sheets fabricated manually. As a result, we confirmed that 49 out of the 50 cell sheets satisfied seven standard criteria including cell morphology, structure, cell number, cell viability, cell purity, and cleanliness. To see if the automatically fabricated cell sheets are equivalent to manually fabricated ones, we performed histological observations of oral mucosal epithelial cell sheets by means of immunostaining and transmission electron microscopy, from which we confirmed that the automatically fabricated cell sheets had differentiated into basal, spinous, and granule layers in the same way as manually fabricated cell sheets.

## 2 MATERIALS AND METHODS

### 2.1 Cell culture

For the automated culture test, we used human oral mucosal epithelial cells manufactured by CELLnTEC (Bern, Switzerland), Lot numbers ES1305033 and MC1507246. These cells were passaged and expanded to prepare the number of cells required for testing. As the culture medium for growth, we used a special culture medium provided with the cell sample for Lot No. ES1305033, while we used an epithelial cell culture medium produced by CELLnTEC for Lot No. MC1507246. A 1% mixture of antibiotics (Nacalai Tesque, Kyoto, Japan) was added to each culture medium. For passaging, we used 0.25% trypsin, 1 mM ethylenediaminetetraacetic acid (EDTA) solution (Nacalai Tesque), and a trypsin inhibitor solution (derived from 0.1% soy beans) (DS Pharma Biomedical, Osaka, Japan). The cells were seeded at 0.4 × 10^4^ cells/cm^2^, cultured for 5 days, passaged, and then cultured for a further 5 days.

For the cell sheet culture, we used a keratinocyte medium (KCM). KCM is a culture medium made from a 3:1 mixture of Dulbecco's modified Eagle medium (DMEM; Merck, Darmstadt, Germany) and F12 (Merck), to which 5% fetal bovine serum (Biowest, Nuaillé, France), 1% mixture of antibiotics (Nacalai Tesque), 1 nM cholera toxin (Wako Pure Chemicals, Osaka, Japan), 2 nM triiodothyronine (Wako Pure Chemicals), 5 μg/ml Insulin–Transferrin–Selenium solution (Thermo Fisher Scientific, Waltham, MA, USA), 5 μg/ml transferrin (Thermo Fisher Scientific), 10 ng/ml epidermal growth factor (Proteinexpress, Chiba, Japan), and 0.4 μg/ml hydrocortisone (Wako Pure Chemicals) are added. In order to fabricate the cell sheets, we used a temperature‐responsive insert manufactured by CellSeed (CellSeed, Tokyo, Japan). After detaching the cultured cells, a cell suspension was prepared in KCM culture medium with a cell concentration of 2.2–2.8 × 10^5^/ml. In the automated cell culture, a suspension of cells was dispensed into seeding bottles with 8.0–10.0 × 10^4^ cells/cm^2^ per 1.5 ml, and 3.4–4.2 × 10^5^ cells per insert, after which the automated cell culture was started. For the manual culture used as a control, we set the inserts into a 6‐well plate, and seeded the inserts with a pipette from a cell suspension. The culture medium was replaced on the 3rd, 5th, 7th, 8th, 9th, 10th, and 11th days. On the 12th day, the culture was terminated and the cell sheets were harvested.

### 2.2 Harvest of cell sheets and measurement of cell numbers/viability

When the culture had been completed, the culture vessels were treated in a 5% CO_2_ incubator at 20°C for 30 min. After that, the cells were washed three times with Hank's balanced salt solution (HBSS; Merck), and the cell sheets were harvested under a stereoscopic microscope. The cell sheets were inspected visually. Then, they were placed in a tube containing a solution of 0.25% trypsin and 1 mM ethylenediaminetetraacetic acid (Nacalai Tesque), cut finely with scissors, and treated at 37°C for 15 min. After dispersing the cells by pipetting, KCM was added and the mixture was passed through a 40 μm cell strainer (BD Biosciences, #352340). The tube was then centrifuged for 5 min at 1,000 rpm at room temperature, resuspended, and stained with trypan blue so that the number of cells and the cell viability could be ascertained.

### 2.3 Measurement of cell purity

The cell suspensions were centrifuged and washed once with 3 ml of a flow cytometer washing solution (BD Bioscience, Franklin Lakes, NJ, USA). After centrifuging at 1,500 rpm for 5 min at 4°C, the samples were incubated for 20 min at 4°C in a fixative (BD Bioscience). After fixation, they were centrifuged at 3,500 rpm for 10 min at 4°C. After washing, one sample was reacted for 1 hr at room temperature with fluorescein isothiocyanate (FITC)‐labeled anti‐pan‐cytokeratin (CK) antibodies (Progen, Heidelberg, Germany), and another was reacted for 1 hr at room temperature with fluorescein isothiocyanate‐labeled anti‐mouse immunoglobulin G2a (IgG2a) antibodies (SC‐2856 Santa Cruz Biotechnology, Santa Cruz, CA, USA). After these reactions, the cells were washed twice in a washing solution and resuspended to measure the pan‐CK positive ratio by flow cytometry.

### 2.4 Hematoxylin and eosin staining and immunostaining

The cell sheets were fixed with 10% neutral buffered formalin solution (Wako, #062‐01661), after which they were embedded in paraffin and stained with hematoxylin and eosin (H&E). We used paraffin blocks for immunostaining. We used Abcam anti‐cytokeratin 3 antibodies [AE5] (Abcam, Cambridge, UK) as the primary antibodies for keratinocyte 3 (CK3), and used Dako anti‐p63 mouse monoclonal antibodies [DAK‐p63] (DakoCytomation, Glostrup, Denmark) as the primary antibodies for p63. As the secondary antibodies, we used BioGenex one‐step polymer‐horseradish peroxidase (HRP) (BioGenex, San Ramon, CA, USA) for both CK3 and p63. The immunostaining method consisted of the following steps: deparaffinization → p63 antigen activation (10 mM pH 6.0 citrate buffer, 121°C, 5 min) → inactivation of endogenous peroxidase (3% aqueous hydrogen peroxide, 5 min) → primary antibody reaction → secondary antibody reaction → DAB staining → nuclear staining → penetration and encapsulation.

### 2.5 Transmission electron microscopy (TEM)

The cell sheets were fixed with 10% neutral buffered formalin solution (Wako Pure Chemicals). Prefixing was performed with 2% glutaraldehyde/0.1 M phosphate buffer at 4°C, followed by postfixing with 2% aqueous osmium tetroxide at 4°C for 3 hr and dehydration processing with 50%–100% ethanol. This was followed by thermal polymerization embedding using EPN812 → preparation of ultra‐thin 90 nm sections using an ultra‐microtome → staining with a two‐part dye consisting of lead/uranyl acetate. Observations were performed using a transmission electron microscope (TEM) with an acceleration voltage of 80 kV.

## 3 RESULTS

### 3.1 Device configuration

**Figure 1:**
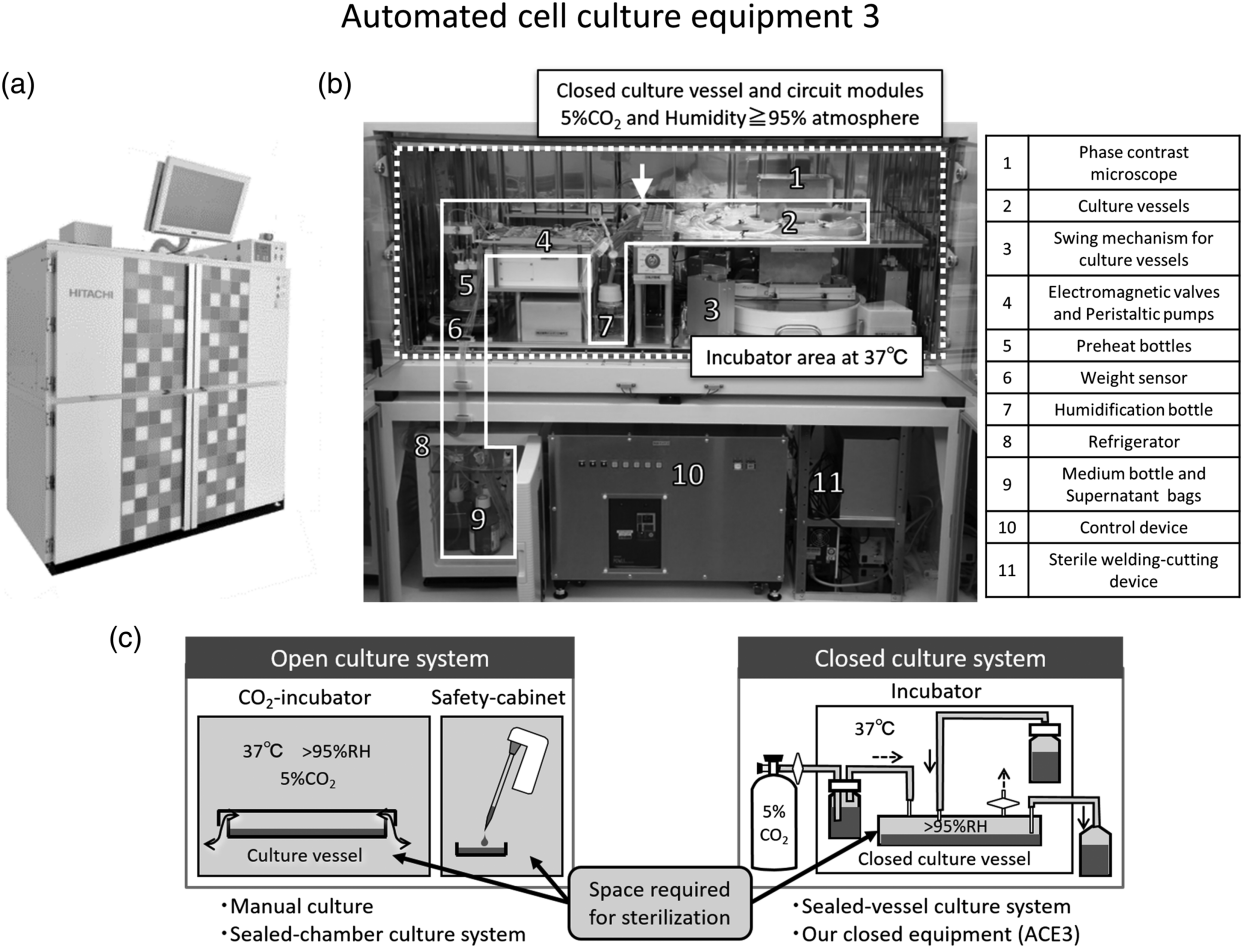
Automated cell culture equipment. (a) External view of the automated cell culture equipment (ACE3). (b) Internal configuration. (c) Difference between open culture system and closed culture system. The incubator area is at the top, and the control area and storage cabinet (including the refrigerator) are at the bottom. The white dotted outline indicates the incubator area where cell culturing is performed at 37°C, but humidified 5% CO_2_ is only supplied in the closed culture vessel and circuit surrounded by the white solid line. The inside of the device, including the shaking mechanism, valves, and pumps, is kept dry. The refrigerator at the bottom is where the culture medium and recovered supernatant can be stored. The equipment also includes a sterile welding‐cutting device for aseptically removing culture vessels and culture supernatant and control device. The open culture system is the conventional culture method and it means a manual culture and sealed‐chamber culture system using robotic arm. Light gray parts indicate spaces that need to be sterilized. The closed culture system that we use requires very little space for cleanliness

An overview of the automated cell culture equipment (ACE3) is shown in Figure 1a,b. The equipment is relatively compact (1,700 mm × 795 mm × 1,580 mm W × D × H), and can be installed in a cell processing facility. It is easy to culture 10 cell sheets in parallel by installing 10 closed culture vessels inside this equipment. Each of the 10 closed culture vessels has independent circuits to maintain the accuracy of the air and liquid feed rates. The CO_2_ concentration in the circuit is maintained at 4.8–4.9%, and the liquid feed accuracy is ±3.2%. The equipment consists mainly of an incubator, a refrigerator, a control box, an uninterruptible power supply, a controller personal computer (PC), and a router PC. The controller PC can be operated remotely via the router PC. The incubator is equipped with a weight sensor, circuit module, and phase contrast microscope. The difference between an open culture system, which uses the conventional culture method, and our closed culture system is shown in Figure 1c. The open culture system requires sterilization of the entire interior of the device during batch changes. On the other hand, the closed culture system requires very little space for cleanliness, and only the closed culture vessel and circuit modules which are sterilized by gamma ray irradiation in advance have to be replaced in this system, making the risk of contamination lower. During culture, the interiors of the closed culture vessel and circuit modules of the ACE3 are filled with a high (95% or more) humidity environment with 5% CO_2_, while the other parts of the equipment interior are filled with the same atmosphere as the outside air. By reducing the humidity inside the main body of the equipment to provide a dry environment, we can reduce the risk of contamination by bacteria and the like, resulting in a lower risk of equipment failure. To keep the interior of the equipment sterile, it also uses an externally driven pinch valve and a peristaltic pump.

The main automated functions of ACE3 are cell seeding, cell culture, gas exchange, medium exchange, and microscopic observation. The microscopic observations can automatically take photographs of each closed culture vessel from multiple fixed points at a frequency set by the user. The user can manually observe and photograph from arbitrary positions in the closed culture vessels or can remotely control these operations.

Here, we should note that there are two major differences from our previous equipment (T. Kobayashi et al., 2013). First, our previous equipment was a semi‐closed system, because the culture vessels were connected to the circuits only at the times of cell seeding and medium exchange. In contrast, it is a completely closed system in ACE3, because the culture vessels were always connected and never disconnected throughout the automated culture. Second, we increased the number of cell sheets that can be cultured at the same time from four to 10, because the transplantation target was expanded from the cornea to the esophagus after endoscopic submucosal dissection. Ten cell sheets are available for inspection the day before transplantation and as spares. Multiple sheets are used when the affected area is large (Ohki et al., 2012).

### 3.2 Closed culture vessel and circuit modules

**Figure 2:**
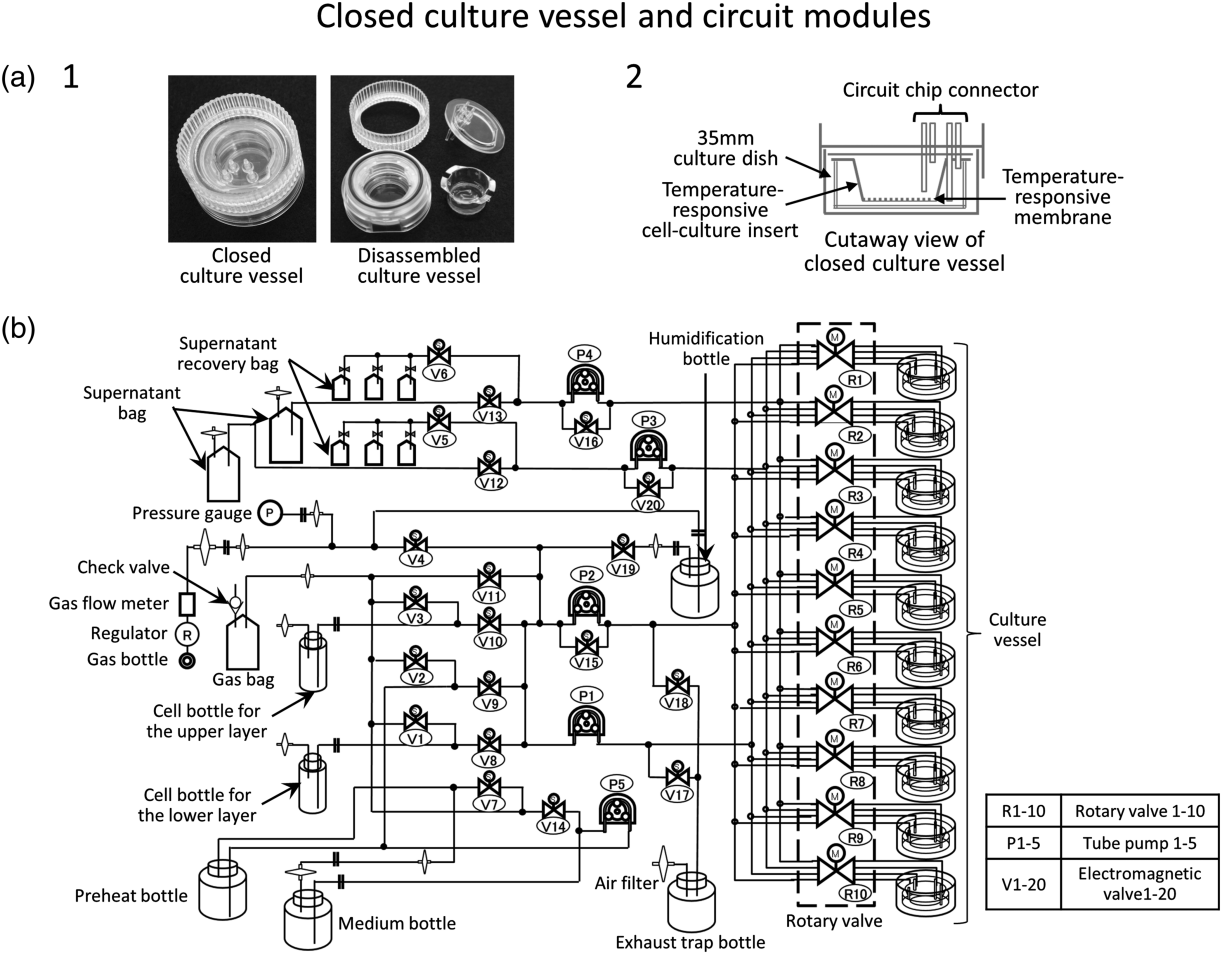
Closed culture vessel and circuit modules. (a‐1) Closed culture vessels in assembled and disassembled states. These are quoted from Hitachi Review (Shu et al., 2016). (a‐2) Cross‐section through a closed culture vessel. The culture dish and temperature‐responsive cell culture insert are set in the vessel, and the culture medium and gas are exchanged via the port. (b) Circuit diagram of the closed system circuit. By switching valves, it is possible to pump the culture medium and gas to any location

The closed culture vessel and circuit modules consist of culture vessels, circuits (tubes), bottles and bags. These are provided as disposable assemblies that can be detached from the equipment and replaced for each culture. This makes it possible to avoid cross contamination between lots. The modules, except for temperature‐responsive cell‐culture inserts, are sterilized by gamma irradiation. The temperature‐responsive cell‐culture inserts are set in the culture vessels of the sterilized modules on a clean bench before they are set on the equipment. Sterile adapters are used to attach them to the equipment. Figure 2 shows a closed culture vessel and circuit module. Figure 2a‐1 shows the appearance of the closed culture vessel before and after assembly. The closed culture vessel is compatible with two‐layer cultures and has a sealed structure enclosing a cell culture insert. A cross‐sectional view of the closed culture vessel is shown in Figure 2a‐2. The culture surface of the cell culture insert is subjected to graft processing with temperature‐responsive polymer poly(N‐isopropylacrylamide) (PIPAAm). When the temperature of the culture vessel is reduced from the culture temperature of 37°C to room temperature (below the phase transition temperature of 32°C), the temperature‐responsive polymer changes the culture surface from hydrophobic to hydrophilic. As a result, the adhered cells are detached, and the cell sheet can be harvested without the use of enzymes that can cause damage to the cells (Elloumi‐Hannachi, Yamato, & Okano, 2010). The closed culture vessel has a total of four ports comprising a supply port and a discharge port for each of the cell culture insert and the 35 mm dish (Figure 2a‐2). For cell sheets cultured on the cell culture insert, 5% CO_2_ gas cylinder is supplied from the port for the 35 mm dish in the lower layer in order to avoid shaking the medium.

In the fully closed circuit, bottles and bags for supplying and discharging the cell suspension, culture medium, and 5% CO_2_ are connected to the culture vessel with tubes, and various sensors for monitoring are attached. The details and an image of the circuit are shown in Figures 2b and S1. The cell suspension, culture medium, and 5% CO_2_ are supplied and discharged using a rotary valve mechanism, solenoid valve, and peristaltic pump (Matsumoto et al., 2019). The culture medium is kept in a refrigerator. When exchanging the medium, the required amount is introduced into a preheating bottle in the incubator and preheated to 37°C before being used to exchange the culture medium. To allow the culture supernatant to be inspected for bacterial contamination, it can be collected in a special bag at any time. The closed culture vessels and culture medium supernatant bags can be aseptically removed by using the installed sterile welding‐cutting device. This equipment has a case composed of heat storing materials especially for transferring culture vessels. Once the culture vessels are in the case, they can be carried to the operating room while maintaining sterility and a temperature of 37°C.

### 3.3 Automated culture tests

To verify the performance of the ACE3, we performed automated culture tests using commercially available human oral mucosal epithelial cells. For these tests, we selected the best lot in which the cells form a cell sheet in a manual culture in advance. We set up an automated cell culture protocol corresponding to the manual culture protocol and seeded the cells automatically in 10 closed culture vessels. After 12 days of automated cell culture, we harvested the cell sheets and evaluated their quality. Seeding was performed with 3.4–4.2 × 10^5^ cells per insert in the automated cell culture, and with 3.4 × 10^5^ cells per insert in the manual culture control.

Our criteria for evaluating the quality of the automatically cultured cell sheets are as follows: (1) the cells should have a dense cobblestone‐like morphology and multiple layers, (2) the cell sheets should be harvested without suffering any damage, (3) there should be at least 1 × 10^5^ cells per sheet, (4) the cell viability should be at least 70%, (5) the epithelial cell purity (pan‐CK positive ratio) should be at least 70%, and the cells must give negative results in (6) sterilization tests and (7) mycoplasma inspections. Table 1 shows the results of five automated culture tests based on these criteria. In each test, 10 sheets were fabricated using the automated cell culture, and five sheets were fabricated manually. The automated cell culture achieved a high yield of 49 out of 50 cultures in the five tests, with the exception of one failure of sheet formation in the third test. Cells in one of 10 cultures in the third test were amplified well to form cobblestone‐like morphology, but after that, they failed to form a cell sheet for some unknown cause.

**Table 1:** Results of fabricating oral mucosal epithelial cell sheets using the automated cell culture equipment

Criteria of human oral mucosa epithelial cell sheet			Results of automated culture tests^a^					
No.	Evaluation item	Standard value	Automated culture/manual culture^b^	1	2	3	4	5
1	Cell property confirmation	Cobblestone‐like morphology and multilayer	Automated culture	Clear	Clear	Clear	Clear	Clear
			Manual culture	Clear	Clear	Clear	Clear	Clear
2	Visual inspection of appearance	No deficit of cell sheet upon removal	Automated culture	Clear	Clear	Clear: 9/10 Not clear: 1/10	Clear	Clear
			Manual culture	Clear	Clear	Clear	Clear	Clear
3	Cell number	1 × 10^5^ cells/sheet or more (×10^5^ cells)	Automated culture	6.31 ± 2.77	4.99 ± 0.41	2.30 ± 0.20	4.32 ± 0.92	4.81 ± 1.24
			Manual culture	3.22 ± 0.98	6.55 ± 1.73	5.04 ± 0.70	4.29 ± 0.34	1.59 ± 0.19
4	Cell viability	>70% (%)	Automated culture	96.5 ± 1.9	96.6 ± 0.6	97.3 ± 0.8	94.9 ± 0.5	95.8 ± 0.7
			Manual culture	96.1 ± 1.4	95.6 ± 1.9	95.8 ± 2.1	94.7 ± 0.7	95.8 ± 2.3
5	Cell purity	Positive rate of pan‐CK>70% (%)	Automated culture	97.8 ± 0.9	97.2 ± 0.6	88.6 ± 2.6	97.0 ± 1.6	94.4 ± 3.6
			Manual culture	98.6 ± 0.9	99.5 ± 0.2	99.6 ± 0.1	98.4 ± 0.1	98.5 ± 0.5
6	Sterilization test	Negative	Automated culture	Negative	Negative	Negative	Negative	Negative
			Manual culture	―	―	―	―	―
7	Mycoplasma inspection	Negative	Automated culture	Negative	Negative	Negative	Negative	Negative
			Manual culture	―	―	―	―	―

a The results for item 1 (multilayer) were obtained from five sheets per test with the automated cell culture, and two sheets per test with the manual culture. For items 3–5, the results show the mean and standard deviation over 5 sheets with the automated cell culture and over 3 sheets with the manual cell culture. For items 6 and 7, we did not perform tests on the manual culture.

b Seeding was performed with 3.4–4.2 × 10^5^ cells per insert in the automated cell culture and with 3.4 × 10^5^ cells per insert in the manual culture control. Manual culturing was performed with the inserts set in a 6‐well plate. In each test, 10 sheets were fabricated with the automated cell culture, and five sheets were fabricated manually.

Depending on the type of analysis, we performed inspections on all or some of the resulting cell sheets. (1) To check the cobblestone‐like morphology during the culture, we observed all 10 of the sheets fabricated automatically and all five of the control sheets fabricated manually. To check the layered structure, we used HE staining to examine five automatically fabricated sheets and two of the manually fabricated control sheets. Quantitative values for (3) the number of cells, (4) cell viability, and (5) epithelial cell purity were calculated as the mean and standard variation over five automatically fabricated sheets and three manually fabricated sheets as a control. In the 49 sheets that were available for testing, we found that the automatically fabricated cell sheets met all the above criteria. Furthermore, they all tested negative for bacteria and mycoplasma. No tests for bacteria or mycoplasma were performed on the manually fabricated cell sheets.

**Figure 3:**
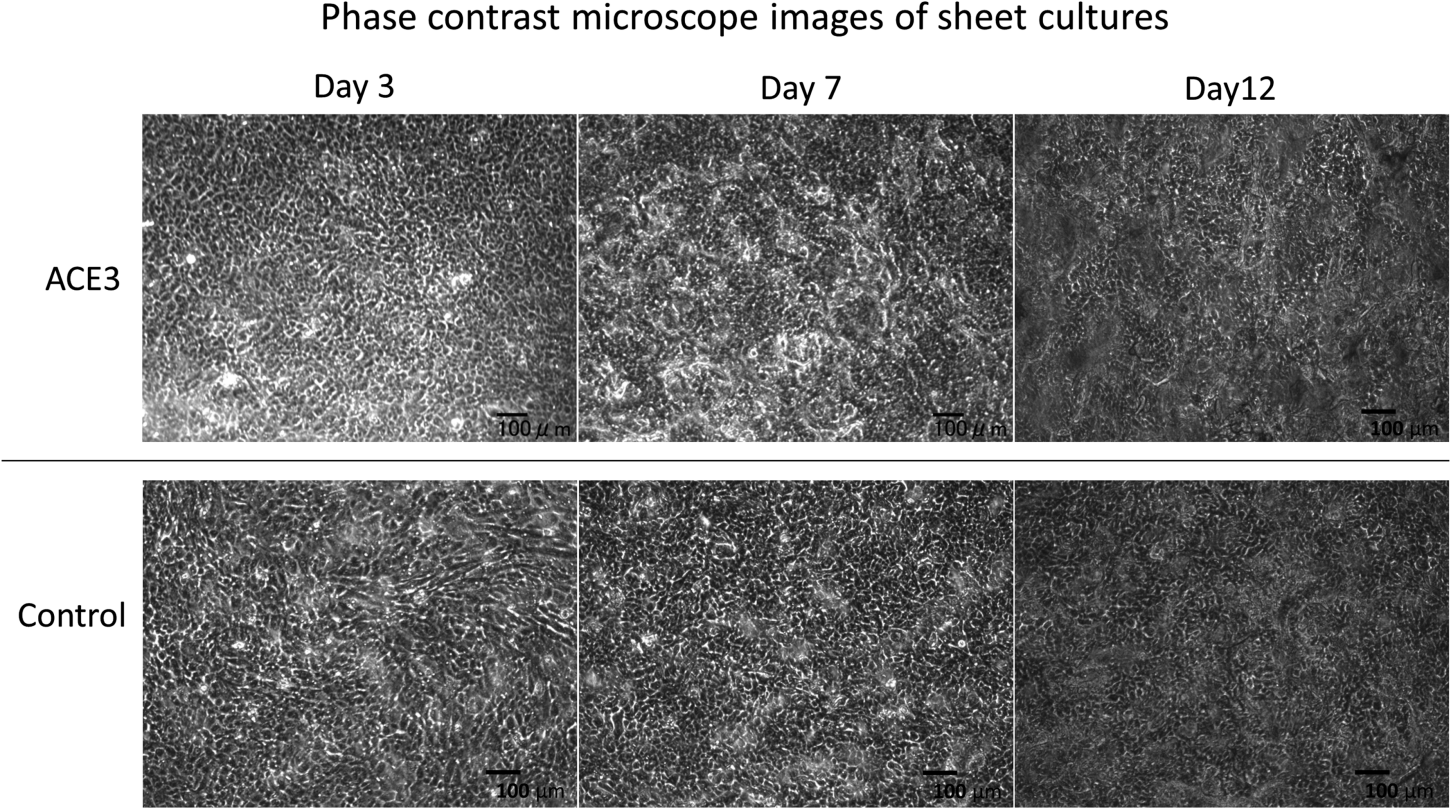
Phase contrast microscope images of sheet cultures. The automated cell culture and manual cell culture control were performed in KCM medium for 12 days. In each test, 10 sheets were fabricated by automated cell culture, and five sheets were fabricated manually. These micrographs were captured during the fourth automated cell culture test. KCM, keratinocyte medium

**Figure 4:**
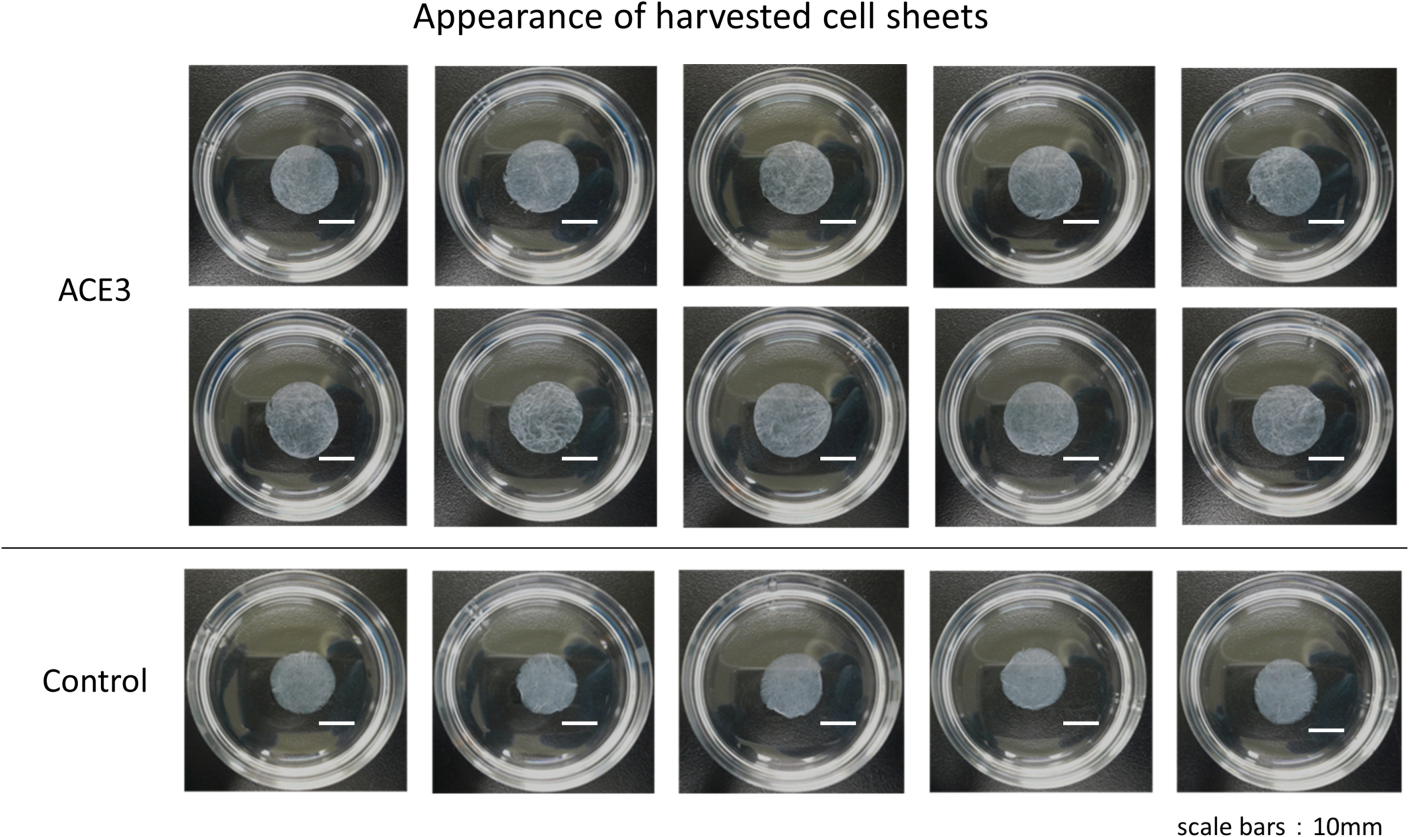
Appearance of harvested cell sheets. The automated cell culture and manual cell culture control were performed for 12 days in KCM medium. In each test, 10 sheets were fabricated by automated cell culture, and five sheets were fabricated manually. This figure shows the results of the fifth automated cell culture test. KCM, keratinocyte medium [Colour figure can be viewed at http://wileyonlinelibrary.com]

Phase contrast microscope images acquired during the automated cell culture are shown in Figure 3. Epithelial stem cells and progenitor cells derived from the basal layer that are used as the source cells grow after adhering to the surface of the culture vessel. They become confluent, resulting in a cobblestone appearance, and then form overlapping layers of cells that are differentiated from the epithelial stem cells and progenitor cells. This process results in the formation of a cell sheet (Hayashi et al., 2010). In both the automated and manual cell culture methods, a cobblestone‐like morphology was confirmed on the 3rd day of culture and the cells formed layers on the 7th day. At the end of the 12th day of automated cell culture, the culture vessel was aseptically removed from the equipment, after which it was incubated at 20°C for 30 min in a 5% CO_2_ before harvesting the cell sheet. The cell sheet is shown in Figure 4. In both the automated and manual cell culture techniques, it was possible to recover a circular cell sheet without any damage.

### 3.4 Histological analysis of cell sheets

**Figure 5:**
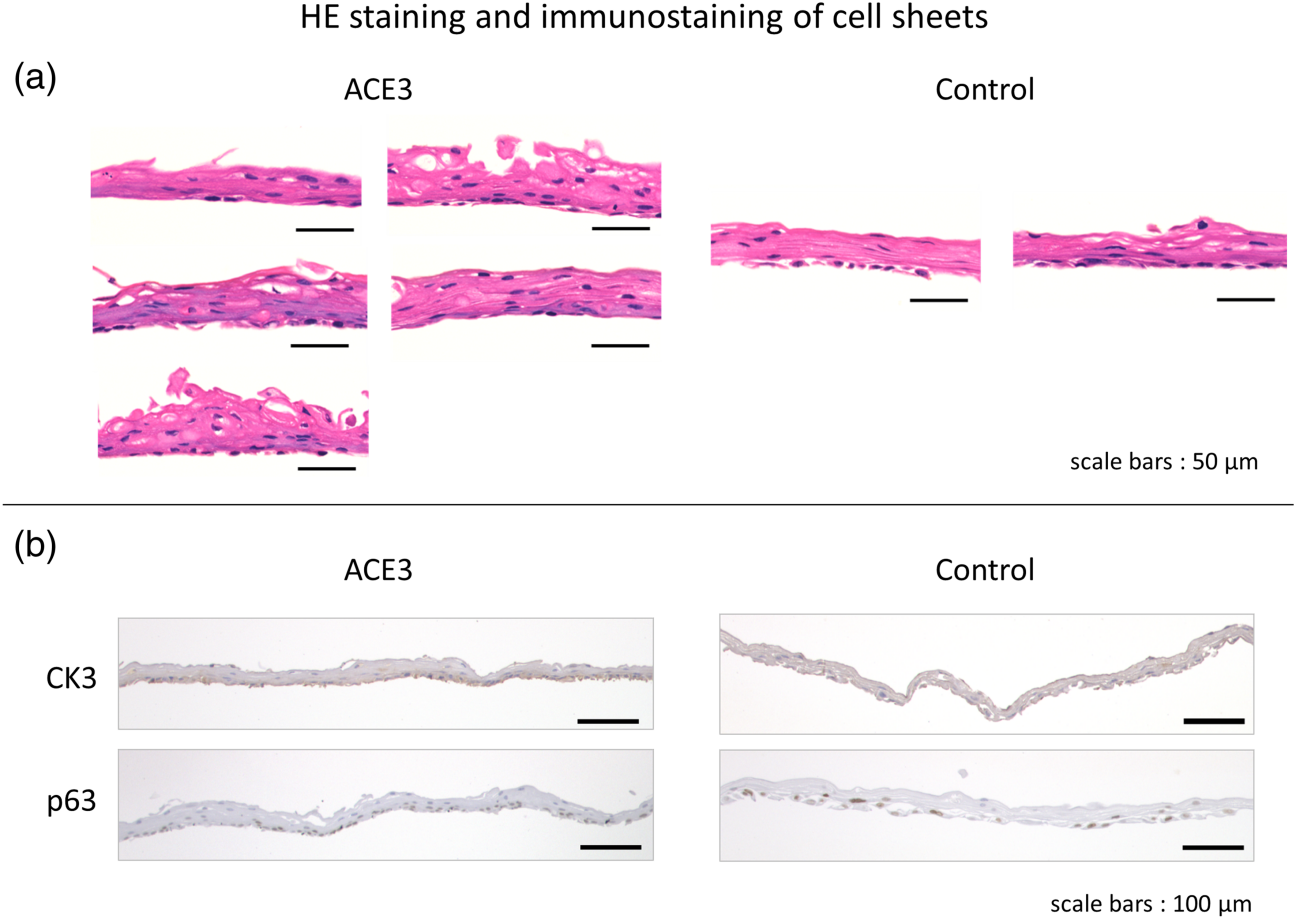
HE staining and immunostaining of cell sheets. HE staining was performed on (a) five samples per test of the automated cell culture, and (b) two samples per test of the manual culture control. The figure of HE staining shows the results of the fourth automated cell culture test. The images of immunostaining were obtained from cell sheets embedded in paraffin blocks using anti‐cytokeratin 3 antibodies and anti‐p63 mouse monoclonal antibodies. The figure of immunostaining shows the results of the fifth automated cell culture test. HE, hematoxylin and eosin [Colour figure can be viewed at http://wileyonlinelibrary.com]

For the histological analysis, we performed HE staining, immunostaining, and transmission electron microscope observations. The results of HE staining are shown in Figure 5a. In HE staining, the cell nuclei are stained purple with hematoxylin, and the cytoplasm is stained red with eosin. We confirmed that epithelial stem cells and progenitor cells derived from human oral mucosal epithelium had differentiated and formed roughly 3–5 layers in cell sheets formed by both automated and manual cultures.

The results of immunostaining with keratinocyte 3 (CK3) and p63 antibodies are shown in Figure 5b. CK3 is a marker for mucosal epithelial differentiation, and p63 is a marker that localizes in the nuclei of epithelial stem cells and progenitor cells (Hayashi et al., 2010; Nishida et al., 2004; Oie et al., 2010). In both the automated and manual cell cultures, CK3 stained the whole cell sheet dark reddish‐brown, thus confirming that CK3 is generally expressed as previously reported. With p63, the nuclei in the basal layer of the cell sheet were stained dark reddish‐brown in both the automated and manual cell cultures, and as previously reported, we confirmed that the basal layer consists of epithelial stem cells and progenitor cells. The CK3 staining was denser in the basal cells of the lower layer, but this is thought to be because the basal cells are smaller and more tightly packed.

**Figure 6:**
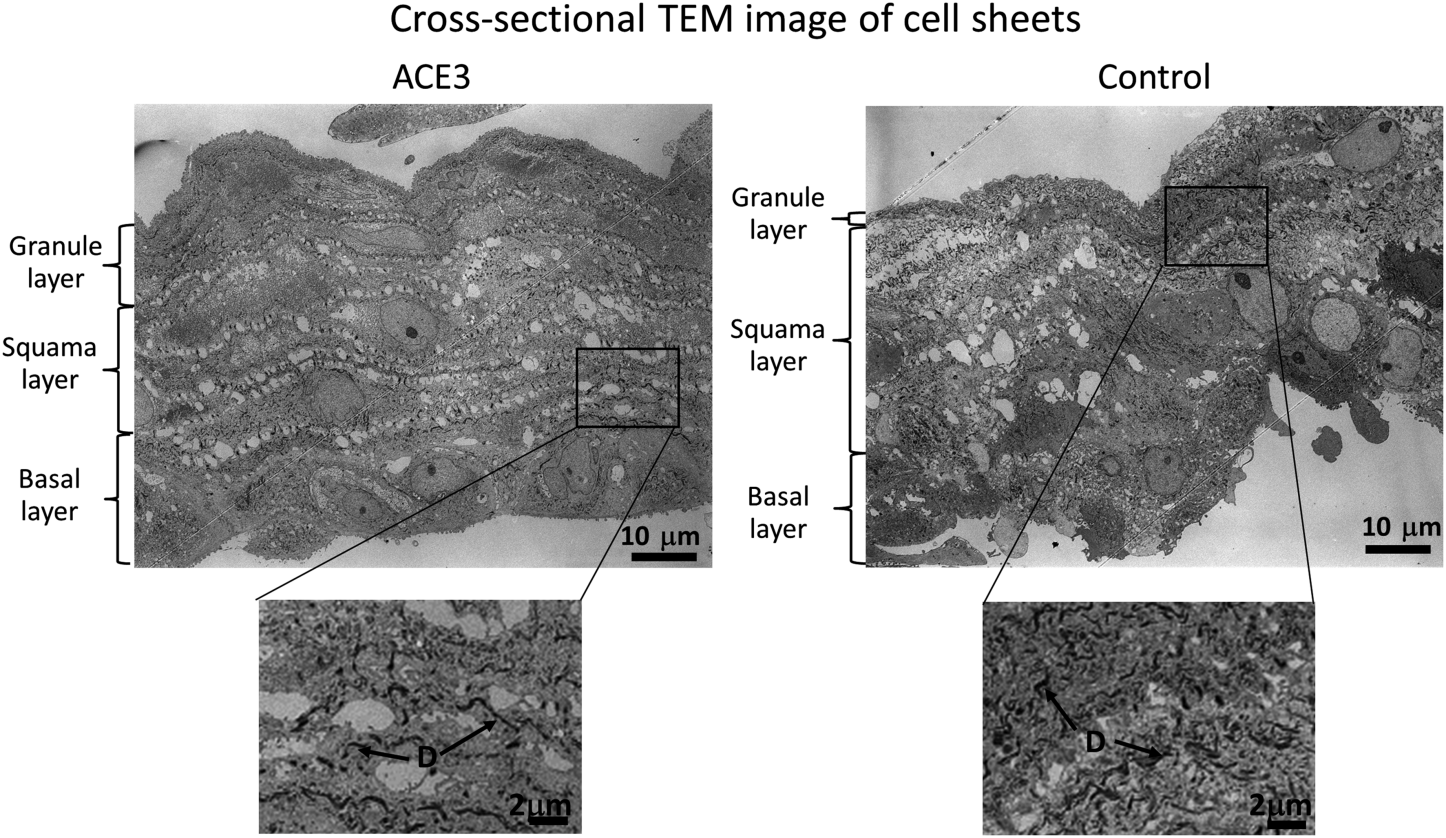
Cross‐sectional TEM image of cell sheets. The basal layer cells are at the bottom, and the apical surface is at the top. Observations were performed using transmission electron microscopy with an acceleration voltage of 80 kV. D, desmosomes; TEM, transmission electron microscope

Figure 6 shows the results of cross‐sectional transmission electron microscope (TEM) observations of the cell sheets. The basal layer cells are at the bottom, and the apical surface is at the top. It is known that oral mucosal epithelial cells differentiate into the following sequence of functional layers: (1) basal cells, (2) spinous cells, (3) granule cells, and (4) keratinocytes (Schoop, Mirancea, & Fusenig, 1999; Tobin, 2006). The basal layer consists of cells that are packed densely in parallel rows at the bottom of the sheet. The spinous layer consists of spinous cells with spinous protrusions, and is characterized by the presence of cell structures called desmosomes at the boundaries between spinous cells and between spinous cells and basal cells. The granule layer consists of flat cells containing granules of keratohyalin. As shown in the enlarged photograph at the bottom of Figure 6, the presence of desmosomes was confirmed in both the automated and manual cell cultures. From these results, we confirmed that the oral mucosal epithelial cells differentiated into three functional layers (i.e., basal, spinous, and granule layers) in cell sheets fabricated by both automated and manual cell cultures. From our histological analysis of the cell sheets by HE staining, immunostaining, and transmission electron microscopy, we confirmed that the cell sheets fabricated with the automated cell culture are equivalent to those fabricated manually.

## 4 DISCUSSION

The manual culture systems are of the open‐culture type. In particular, sealed‐chamber culture systems using robotic arms are of this type. A number of automated cell culture systems have been developed since 2000 and many of them are of the sealed‐chamber type. Because the sealed‐chamber culture system requires sterilization of the entire interior of the device during batch changes, its operation is time‐consuming. On the other hand, the sealed‐vessel culture system we use only has a small volume that requires sterilization and has low risk of contamination (Kino‐oka & Taya, 2009). In our equipment, the volume of the closed culture vessel and circuit modules that require sterilization amounts to only 5 L, and changing batches is easy because we only need to replace the sterilized culture vessel and circuit modules. Low risk of contamination and ease of operation are very important advantages for the automated culture systems.

As a result of automated culture tests using human oral mucosal cells, we succeeded in fabricating epithelial cell sheets that satisfy the criteria for cell sheets fabricated manually, and we confirmed that cell sheets fabricated by automated cell culture are histologically equivalent to those fabricated manually. We also succeeded in culturing cell sheets five times in succession and were able to show that the automated cell culture equipment is capable of stable production of cell sheets. Automated cell culture has the advantage that it is unnecessary to extract the culture vessel from the incubator in order to perform microscope observations or exchange the culture medium. As a result, it causes smaller changes in temperature and CO_2_ concentration than manual culture, and provides a better culture environment.

This report compared results for an automated culture and those for a manual culture. When the manual culture did not go well, the automated culture did not go well either. The cell sheet fabricated by automated cell culture sometimes did not reach the quality of the manually cultured cell sheet. The automated equipment seeds, supplies gas, and feeds in the medium via dedicated tubes for each closed culture vessel. It is conceivable that the environment of the closed system may cause physical damage to the cells. By performing cell seeding, gas supplying, and medium feeding via tubes in a closed system to minimize physical damage to cells, it should be possible to perform culturing more stably than with manual techniques.

We are also studying the use of this automated culture equipment for the fabrication of cell sheets with different cell types. If we can fabricate cell sheets of various types, then it should be possible to increase the use of cell sheets in regenerative medicine.

Expertise in manual cultures is currently needed for making regenerative medicine products; this is an obstacle to the popularization of regenerative medicine. If automated cell culture techniques can be used to mass produce cell sheets at lower cost with stable quality, more patients could have access to regenerative medicine therapies.

## CONFLICT OF INTEREST

Teruo Okano is a shareholder and the chairman of the Scientific Advisory Board of CellSeed Inc. Tatsuya Shimizu is a shareholder and a member of the Scientific Advisory Board of CellSeed Inc. Masayuki Yamato is a shareholder of the Scientific Advisory Board of CellSeed Inc. Tokyo Women's Medical University received research funding from Hitachi Ltd. and CellSeed Inc.

## AUTHOR CONTRIBUTIONS

A. Nishimura, R. Nakajima and R. Takagi cultured and analyzed the cell sheets. A. Nishimura wrote the manuscript with support from S. Takeda and H. Hanzawa. G. Zhou, D. Suzuki, M. Kiyama, T. Nozaki, T. Nakamura, M. Sugaya, K. Terada, Y. Igarashi and H. Hanzawa developed the equipment. T. Owaki, R. Takagi, T. Takahara and S. Nagai applied the cell culture protocol and evaluation criteria for clinical research. T. Okano, T. Shimizu, M. Yamato and S. Takeda supervised the work. T. Shimizu and S. Takeda approved the final draft.

## Supporting information

Fig S1. Image of detachable closed culture vessel and circuit modules
