# Supplementary figures and images for: Fabrication of tissue‐engineered cell sheets by automated cell culture equipment
Source: J Tissue Eng Regen Med. 2019 Nov 14;13(12):2246–55. doi: 10.1002/term.2968 (PMC6972683; doi:10.1002/term.2968)

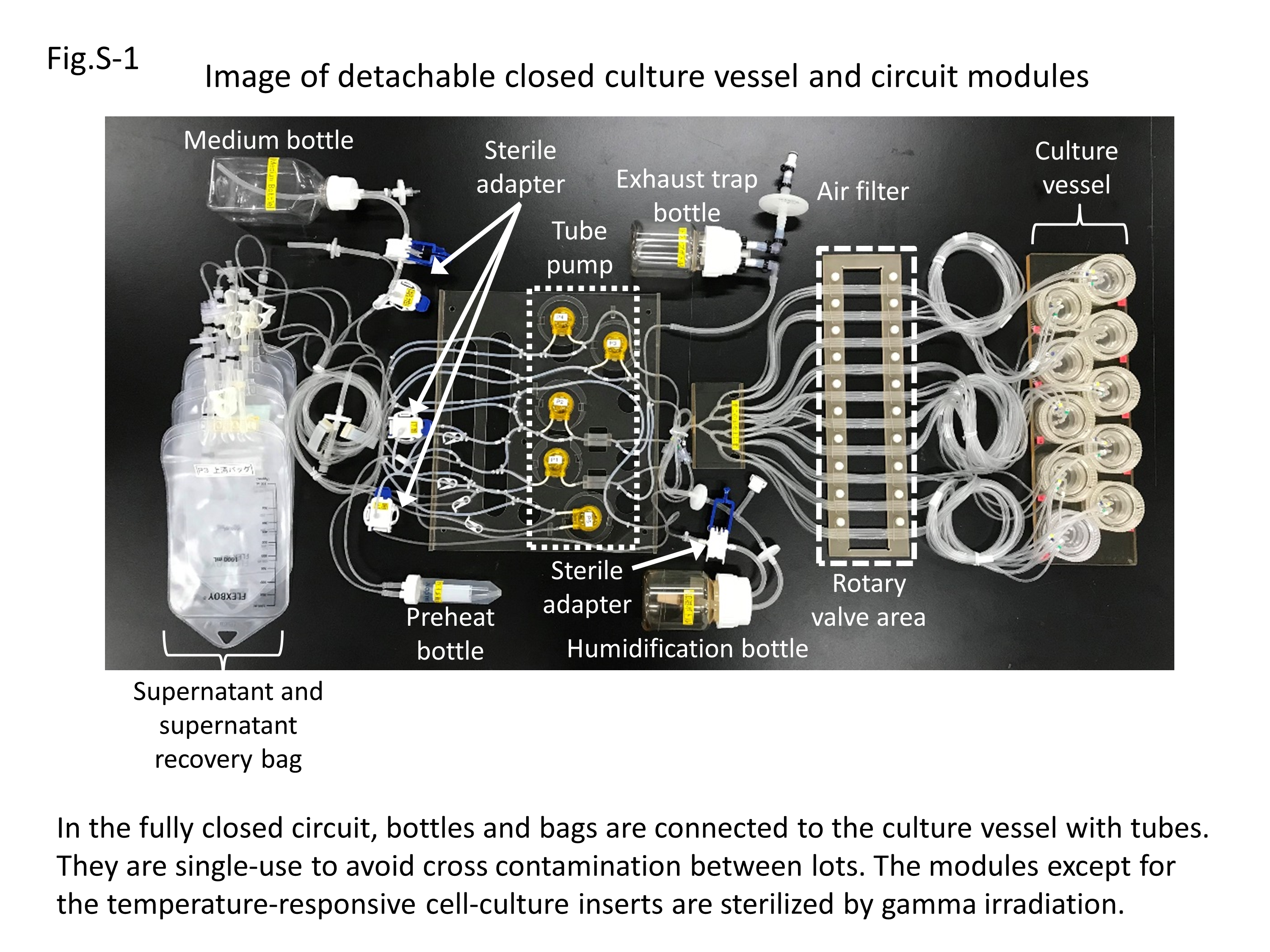

Supplement: Supplementary file 1 — Fig S1. Image of detachable closed culture vessel and circuit modules [file TERM-13-2246-s001.tif]
